# Gastrointestinal Parasites in Non-Human Primates in Zoological Gardens in Northern Italy

**DOI:** 10.3390/ani14172607

**Published:** 2024-09-07

**Authors:** Erica Marchiori, Lucia Bono, Laura Voltan, Giorgia Dotto, Cinzia Tessarin, Federica Marcer

**Affiliations:** 1 Department of Animal Medicine, Production and Health, University of Padova, viale dell’Università 16, 35020 Legnaro, Italy; erica.marchiori@unipd.it (E.M.); giorgia.dotto@unipd.it (G.D.); cinzia.tessarin@unipd.it (C.T.); 2 Parco Faunistico Cappeller, Via Kimle, 12, 36050 Cartigliano, Italy; luciabono150@gmail.com; 3 Parco Faunistico Valcorba, via Val Corba 18, 35020 Pozzonovo, Italy; lauravoltan20@gmail.com

**Keywords:** non-human primates, *Giardia*, *Blastocystis*, helminths, zoological gardens

## Abstract

**Simple Summary:**

Non-human primates, due to their close taxonomic relationship with humans, host the highest diversity of parasites with zoonotic potential. In zoological gardens, the presence of helminths and protist parasites with a direct lifecycle is virtually unavoidable; biosecurity measures are of the utmost importance to control their spread and environmental load and avoid transmission to staff and visitors. In this study, we investigated the population of non-human primates in two zoos in Northern Italy to evaluate gastrointestinal parasite diversity and their zoonotic potential. The highest prevalence was registered for protist taxa, i.e., *Giardia* and *Blastocystis*. Proof for the transmission of parasites from synanthropic rats to the animals in the collection was also provided.

**Abstract:**

Non-human primates (NHPs) host a variety of helminth and protist parasites that are able to cause infection in humans. Gastrointestinal parasites in NHPs living in two zoological gardens of Northern Italy were studied. An total of 96 faecal pools were collected from 26 groups of NHPs. The mini-Flotac method was applied to fecal samples to detect gastrointestinal helminthiases, while the detection of the protists *Cryptosporidium* spp., *Blastocystis* sp. and *Giardia duodenalis* was performed by targeting SSU rRNA through nested PCR and real-time PCR; they were further studied by sequencing the same gene for *Blastocystis* and βgiardine and triosephosphate isomerase (TPI) genes for *Giardia*. Twenty-two out of the 96 examined fecal pools (22.9%) were positive for one or more helminth species, including *Hymenolepis diminuta*, Trichurid, Capillariid and Strongylid eggs. All samples were negative for *Cryptosporidium* spp., while 16/26 (61.5%) animals were positive for *G. duodenalis* in the real-time PCR; the sequences obtained assigned them all to sub-assemblage BIV. *Blastocystis* sp. was detected in 22/26 of the NHPs (84.6%); molecular analyses attributed the isolates to ST 4, allele 92. Analyses of the feces of sympatric rats revealed the presence of the same allele, as well as of *Hymenolepis diminuta* eggs, raising concern about their role as parasite reservoirs in the facilities.

## 1. Introduction

The close phylogenetic relationship between humans and non-human primates (NHPs) is the basis of the potential transmission of all classes of pathogens among the two. Biosecurity measures are of the utmost importance in zoological gardens to avoid the spreading of pathogens with zoonotic potential to the environment, given the close proximity between the animal collection and their human caregivers, and potentially, visitors.

Monoxenous parasites, here including both helminthic and protozoan species, greatly prevail in zoological gardens, facilitated by fecal–oral transmission and the accumulation of their infective forms in the environment of the enclosure. The spreading of protozoan infections is facilitated by the lower infecting dose and brief prepatent period. An assessment of the zoonotic risk coming from the contact between NHPs and humans has been possible with the development of fine molecular tools that allow the definition of isolates beyond species, assemblages or subtypes, the identification of zoonotic and non-zoonotic strains, and the determination of the likelihood of transmission from animals to humans or vice versa. To date, four species of *Cryptosporidium*, including *Cryptosporidium hominis*, *Cryptosporidium parvum*, *Cryptosporidium felis* and *Cryptosporidium muris*, have been detected in NHPs in captive or wild conditions [1,2,3,4], with the bulk of the isolates from captive NHPs belonging to zoonotic strains of *C. hominis* [3,4,5]. *Giardia duodenalis* is considered a species complex with at least eight distinct genotypes/assemblages, designated as A–H. The couple A+B assemblages make up 95% of all isolates in both NHPs and humans, while assemblage E is less frequent but not rare in both groups [6,7,8]. The study of human and animal isolates through multilocus analysis has defined the existence of subtypes within assemblage A and B that are more adapted to one of the two groups, but the great bulk of the strains are shared between humans and NHPs, demonstrating possible transmission between the two [8,9,10]. *Blastocystis* is the most commonly found eukaryotic protist in the intestinal tract of humans and captive NHPs in surveys worldwide. At least 28 subtypes, named ST1-ST17, ST21 and ST23-32, have been recognized within the genus, based on polymorphism at the small subunit ribosomal RNA gene. A different host tropism is typical to each subtype, with a relevant overlap in the host spectrum among subtypes, including humans among animal hosts. Though it is now quite clear that certain subtypes are more often associated with disease in humans, causing IBS and urticaria, *Blastocystis* in wildlife and captive animals is often characterized as asymptomatic [11]. 

Strongylid nematodes are among the most commonly reported gastrointestinal parasites found in wild primates [12]. Primate species can be infected with several nematodes of the suborder Strongylida, such as hookworms (*Ancylostoma*, *Necator*), *Trichostrongylus*, *Ternidens*, and *Oesophagostomum*, whose eggs cannot be distinguished with certainty by microscopy alone. 

The available data on the parasitofauna of NHPs in Italy are mainly related to zoological gardens located in central and southern Italy. With this survey, the prevalence of gastrointestinal parasites in captive NHPs in two zoological gardens in Northern Italy is evaluated, focusing on helminthic and protist taxa, with the aim of evaluating potential zoonotic transmission and informing correct prophylactic measures. The occurrence of parasitic taxa in rats living in sympatry in the zoological gardens was also investigated to reveal their eventual epidemiologic role in parasite transmission to NHPs. 

## 2. Materials and Methods

### 2.1. Sampling 

Fecal samples of non-human primates were collected from two zoological gardens (zoological garden 1 = ZG1; zoological garden 2 = ZG2) in Northern Italy. The species of NHPs included in the sampling belonged to the four major taxonomic groups, namely New World Monkeys (Plathyrrini, *n* = 11), Lemurs (Lemuridae, *n* = 8), Old World monkeys (Cercopithecidae, *n* = 6 groups) and apes (Hominoidea, *n* = 1) (overall animal groups *n* = 26) (Table S1). Each group was hosted in the same enclosure for the entire duration of the project, with both indoor and outdoor accommodations available. The sampling was repeated in each enclosure four times in a one-year period, precisely in the months of April (*n* = 24 samples), July (*n* = 24), October 2021 (*n* = 23) and January 2022 (*n* = 25), finally collecting 96 samples overall from 26 animals or groups of animals hosted in the same enclosure (11 from ZG1 and 15 from ZG2, respectively). Eight samples were missed due to logistic constraints. Sampling was conducted for 3 consecutive days in each enclosure to overcome the intermittent excretion of *Giardia* and *Cryptosporidium* sp. and the three-day samples were pooled in a unique sample. 

Treatments were carried out on the animals as follows: ZG1: fenbendazole or albendazole after the 1st, 2nd and 3rd sampling; ZG2: ivermectin and fenbendazole after the 3rd sampling.

Additionally, the feces of free-living sympatric rats were also collected from the two zoos, in the close vicinity of cages or within them, at the end of the sampling period. Twelve samples, representing different areas of the two zoos (9 for ZG1 and 3 for ZG2), were overall collected. 

After collection, the stool samples were stored at +4 °C before analyses and transferred to the Laboratory of Parasitology and Parasitic Diseases of the Department of Animal Medicine, Production and Health, Padova University. 

### 2.2. Traditional Copromicroscopic Analyses

A quali-quantitative copromicroscopic analysis was performed on a 2 g aliquot from each sample, targeting helminth eggs and *Giardia* spp. cysts, by means of the MiniFlotac technique [13], using a 1350 high-gravity solution (sodium nitrate and sucrose). For the research of larvae of bronchopulmonary nematodes, a Baermann technique was additionally employed on all samples, using a further 4–5-g aliquot and allowing the sedimentation of eventually present larvae for 24 h [14]. The eggs and cysts were searched using an optic microscope at 100× and 400×, respectively, identified following guidelines in the literature to the lowest taxonomic level possible [15,16]; these were counted and quantified as eggs/cysts per gram of feces (EPG/OPG). A 50 g aliquot of each fecal sample was then subject to centrifugation to determine the concentration of protozoan elements [17]; this was to increase the sensitivity of traditional and molecular methods targeting *Crytposporidium* spp., *Giardia* and *Blastocystis* spp. More in detail, each stool specimen was homogenized in distilled water, filtered through gauzes into a 50 mL tube and centrifuged at 900× *g* for 30 min. A faecal smear was obtained from the sediment, stained with the modified Ziehl–Neelsen method [18] and observed under a light microscope at 1000× magnification for the research of *Cryptosporidium* spp. oocysts. Additionally, a 2–5-g aliquot of the sediment was frozen at −20 °C for the molecular research of protist parasites.

### 2.3. Molecular Analyses

Molecular research of *Cryptosporidium* spp., *Giardia* and *Blastocystis* was performed on each sample. All primers and probes used are reported below in Table 1 and Table 2. DNA from the stool specimens was extracted using a QIAampDNA Stool Mini Kit (Qiagen, Valencia, CA, USA), following the manufacturer’s instructions. 

Nested PCR targeting the 18S rDNA gene was used for the detection of *Cryptosporidium* spp. following Miller et al. [19]. A qPCR assay was applied for the detection of *Giardia duodenalis*, following Verweij et al. [20]. Positive samples were further studied for their genotype by amplifying fragments covering β-giardine (BG) and the Triophosphate Isomerase (TPI) genes by nested PCR following Lalle et al., 2005 and Sulaiman et al., 2003 [21,22]. A TaqMan qPCR assay was used for detecting *Blastocystis* STs targeting the rDNA 18S gene, following Stensvold et al., 2012 [23]. Positive samples were further characterized into subtypes using the PCR protocol outlined by Scicluna et al., 2006 [24], using the pan-*Blastocystis* primer RD5 and the universal eukaryotic primer BhRDr targeting the barcode region of the 18S rRNA gene. 

The PCR products were visualised using UV light on a SYBR Safe DNA-stained 1% agarose gel. Subsequently, all the secondary PCR products were purified by ExoSAP-IT™ PCR Product Cleanup Reagent (Applied Biosystems, Thermo Fisher Scientific, Milan, Italy) and sent for sequencing at Macrogen Europe (Milan, Italy). The consensus sequences were assembled with the software ChromasPro version 2.4.3 (Technelysium Pty Ltd., South Brisbane, Australia). The sequences were compared with the non-redundant database available in the GeneBank^®^ database using the software BLAST (Blast+2.14.0) [25] and PubMLST (www.pubmlst.org/species-id, accessed on 10 August 2023) [26].

The sequences of *Giardia duodenalis* were attributed to their subassemblages using previously defined references (TPI gene: AF069561—sub-assemblage BIII; AF069560—sub-assemblage BIV; BG gene: AY072726—sub-assemblage BIII; AY072726—sub-assemblage BIV) [8]. 

The sequences obtained from the selected strains isolated in this study have been deposited in the NCBI database.

**Table 1:** Primers used in PCR reactions targeting *Cryptosporidium* spp., *Giardia* spp. and *Blastocystis* sp.

		Primer F (5′-3′)	Primer R (5′-3′)	Amplicon Size (bp)
***Cryptosporidium* spp. [16]**				
18S	1st round	C1F TTCTAGAGCTAATACATGCG	C1R CCC TAA TCT TTC GAA ACA GGA	1325
	2nd round	C2F GGAAGGGTTGTATTTATTAGATAAAG	C2R AAGGAGTAAGGAACAACCTCCA	850
***Giardia* spp. [18,19]**				
β-giardine		G7 AAGCCCGACGACCTCACCCGCAGTGC	G759 GAGGCCGCCCTGGATCTTCGAGACGAC	753
		βGiar-F GAACGAACGAGATCGAGGTCCG	βGiar-R CTCGACGAGCTTCGTGTT	511
TPI		F1 AAATIATGCCTGCTCGTCG	R1 CAAACCTTITCCGCAAACC	605
		F2 CCCTTCATCGGIGGTAACTT	R2 GTGGCCACCACICCCGTGCC	530
***Blastocystis* [21]**				
18S		RD5 ATCTGGTTGATCCTGCCAGT	BhRDr GAGCTTTTTAACTGCAACAACG	600

**Table 2:** Primers used in qPCR reactions targeting *Giardia* spp. and *Blastocystis*.

	Primer F (5′-3′)	Primer R (5′-3′)	Probe (FAM-5′- 3′-TAMRA)
***Giardia* spp. [17]**			
18S	*Giardia* F GACGGCTCAGGACAACGGTT	*Giardia* R TTGCCAGCGGTGTCCG-	CCCGCGGCGGTCCCTGCTAG
***Blastocystis hominis* [20]**			
18S	Blasto FWD F5	Blasto R F2	Probe (FAM-5′-MGBNFQ)
	GGTCCGGTGAACACTTTGGATTT	CCTACGGAAACCTTGTTACGACTTCA	TCGTGTAAATCTTACCATTTAGAGGA

### 2.4. Statistical Analyses

Differences in the occurrence of parasite taxa along the seasons were tested using the using the ꭓ2 test, setting significance at *p* > 0.05. Statistical analyses were performed using the online software EpiTools (AusVet2024), available from AusVet Animal Health Services https://epitools.ausvet.com.au/ (accessed on 28 August 2024). 

## 3. Results

### 3.1. Copromicroscopic Analyses 

**Figure 1:**
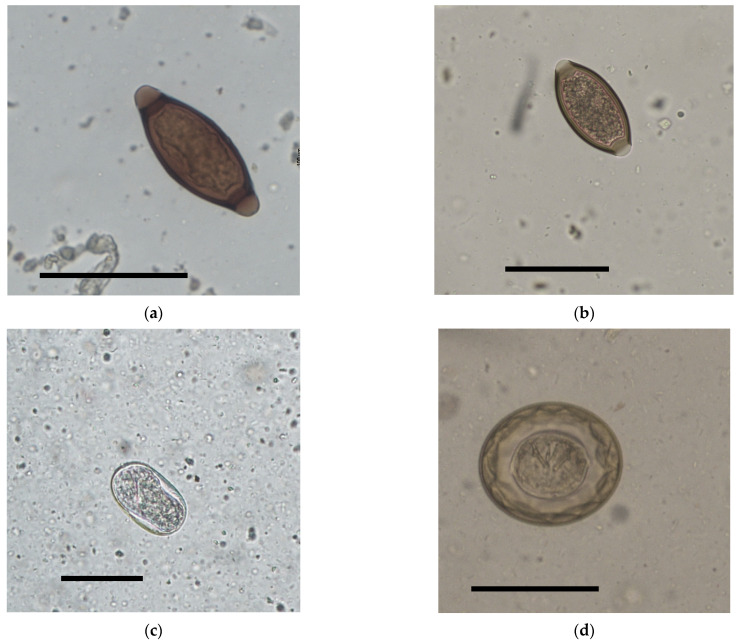
Light microscope pictures of parasite eggs recovered from fecal samples of NHPs: *Trichuris* spp. (**a**) *Lemur catta*, Capillarinae Gen. spp. (**b**) *Colubus guereza*, *Strongyloides* spp. (**c**) *Lemur catta* and *Hymenolepis diminuta*. (**d**) *Callithrix argentata*. Scale bar = 50 ηm.

Twenty-two of the 96 fecal samples (22.9%), belonging to 11 NHPs, were positive for helminth eggs; seven animal groups showed more than one positive sample throughout the sampling period. Eight animals had a co-infection with two or more parasitic taxa. Six helminths and one protozoan parasite were identified, with a higher prevalence of nematode eggs (*Trichuris* spp.: *p* = 10.4%, *Capillariinae* Gen. spp.: *p* = 9.3%, strongylids: *p* = 6.2%, *Strongyloides* spp.: *p* = 2.1%), followed by cestode eggs (*Hymenolepis diminuta*: *p* = 2.1%) and cysts of *Giardia* spp. (1.04%) (Table 3, Figure 1). 

Helminth eggs were observed in the feces of New World Monkeys (6/11), Old World monkeys (2/6) and Lemurs (2/8), while no parasitic elements were detected in the stool of apes. Low parasitic burdens were observed, with the exception of a few samples that were highly positive for strongylid eggs (*Capillariinae* Gen. spp. EPG: 5–90; *Trichuris* spp. EPG: 5–170; strongylid EPG: 10–1120; *Strongyloides* spp. EPG: 35–185; *H. diminuta*: 30–170). 

No gastrointestinal symptoms were observed in parasitized animals during the investigation period. All samples of rat feces were positive for at least one helminth species, namely *Capillariinae* Gen. spp. (12/12, *p* = 100%, EPG: 15–225) and *H. diminuta* (7/12, 58.3%; EPG: 15–800).

### 3.2. Molecular Analyses

Overall, 32/96 samples from NHPs (33.3%) tested positive for *Giardia duodenalis* when the qPCR assay was performed; these belonged to 16/26 animal groups, 10 of them showing more than one positive sample throughout the sampling period. Clear sequences of β-giardine (BG) and triosephosphate isomerase (TPI) genes were retrieved from three and four samples, respectively (BG accession numbers: PQ007548—PQ007549—PQ007552; TPI accession numbers: PQ007553—PQ007555—PQ007557—PQ007558), overall belonging to isolates from six animal groups belonging to four species (3 *Lemur catta*, 1 *Colobus guereza*, 1 *Saguinus oedipus*, 1 *Callithrix jacchus*); none of the isolates could be sequenced at both loci. All isolates were classified as *G. duodenalis* sub-assemblage BIV from alignment with reference sequences (all identities > 99.7%). *Blastocystis* sp. was detected by qPCR in 42/96 samples (43.7%), belonging to 21/26 animal groups, of which 10 had repeated positivity throughout the sampling period. Eight positive samples were suitable and submitted for sequencing, but only eight sequences of good quality were retrieved; these were from *Saguinus tripartitus* and *Varecia rubra*, respectively (Table 3) (accession numbers: PP952159—PP952160). A comparison with the public database PubMLST assigned both the isolates to ST4, allele 92 (length of nucleotidic region: 546 and 615 bp, query coverage 99.9% and 100%). 

One of the rat fecal samples tested positive for *Blastocystis* with PCR, and sequences of good quality were obtained (accession number: PP952161); a comparison with the PubMLST database attributed the isolate to ST4, allele 92 (length of nucleotidic region 615 bp, query coverage 100%). 

A higher prevalence of helminthiases was observed in spring and autumn, while the prevalence of *Giardia* and *Blastocystis* spp. was higher in summer and autumn, respectively. Nevertheless, no statistically significant difference in prevalence was found among the four seasons, neither for helminthic infections nor for *Giardia* or *Blastocystis* (*p* > 0.05).

## 4. Discussion

The permanence of specific animal groups in a given enclosure, sometimes for life-long periods, may lead to the accumulation of the infective forms of parasites in the environment, facilitating the spread of parasites, especially for monoxenous species. In this survey, eggs attributable to four helminth taxa (*Capillariinae* Gen. spp., *Trichuris* spp., strongylids, *H. diminuta*) and two protist genera (e.g., *Giardia* and *Blastocystis*) were isolated from captive non-human primates in two zoological gardens in Northeastern Italy, representing the first report of its kind in the area. 

The prevalence of helmithiases in this survey is lower compared with other studies in zoological gardens of Europe [14,27] and Italy [28]. Among the parasite taxa reported, all of them, except the cestode *H. diminuta*, present a direct life cycle. Fecal–oral transmission parasites are among the most common found in wild animals kept in confined environments. Considering also the social habits of NHPs [29], despite the use of control and treatments, the radical elimination of parasitic elements excreted by the feces is challenging, if not impossible [14,30,31]. In this research, treatments with albendazole/fenbendazole/ivermectin were not efficient in the complete eradication of helminth infection in the enclosures, with the treated animals being positive again at the successive samplings. Despite the associated daily cleaning routine, the resistance of envinronmental parasitic forms might account for the persistence of helminthiases in the captive NHPs.

The cestode *H. diminuta* is primarily a rodent parasite, with occasional reports in other mammals, including NHPs and humans [32,33]. In Italy, it is rarely reported in humans, dogs and pet squirrels [34,35,36]. In this study, *Trichuris* spp. and *Capillariinae* Gen. spp. were the most frequently detected helminths in copromicroscopic analyses. *Trichuris* spp. inhabits the cecum and colon of a broad range of mammals and has a cosmopolitan distribution. In zoological gardens of Europe, *Trichuris* is also one of the most common parasite genera detected in NHPs [31]; in Italy, it was reported in *L. catta*, *Papio cynocephalus*, *Eulemur albifrons, Macaca fuscata* and *Chlorocebus aethiops* in captivity [28,31]. Recent molecular and morphological studies suggest that *T. trichiura*, the only species traditionally thought to infect primates, may actually be considered as a species complex with several sibling/cryptic species, showing different host specificities [37,38]. The phylogenetic analysis performed on both mitochondrial and nuclear markers discriminated between two clades within *T. trichiura*, with some subclades including isolates from both humans and NHPs [38,39,40]. In this study, molecular characterization was not attempted on helminth eggs. As morphological discrimination among clades is unfeasible, at least from eggs, zoonotic risk cannot be excluded in this context. 

*Capillariinae* eggs, observed in this survey in feces of OWM, NWM and rats, had previously been reported via copromicroscopic exam in capuchin monkeys in South America [41] and recently in *Sapajus paella* in a zoological garden in central Italy [31]. *Capillaria brochieri* n. sp. was described in the intestine of *Pan paniscus* with diarrhea in Zaire [42]. *Capillaria hepatica* (syn. *Calodium hepaticum*), a potentially zoonotic species, was histologically described in a retrospective study in the liver samples of primates from a zoological collection in the UK [43] and previously in wild *Gorilla gorilla* in Rwanda; in both cases, the most likely source of infection was judged to be rodents, such as rats, representing the most typical hosts for this species [44]. Despite the difficulties associated with DNA isolation and/or PCR inhibitors, the use of molecular testing could be useful and desirable for identifying species and elucidating the zoonotic potential [31]. 

Such a high prevelance of members of Trichuroidea in this and other surveys may be partially explained by the inefficiency of commonly used anthelminthic drugs against the two genera *Trichuris* and *Capillaria*, as well as by the high resistance of their eggs in the environment.

In this survey, strongylid and *Strongyloides* eggs were observed only in NWM and lemurs. *Oesophagostomum* sp. eggs, strongylid and Trichostrongylid eggs and strongyliform larvae were observed in copromicroscopic analyses of NHPs in zoological gardens in Italy [28,31,45]. Gastrointestinal strongylids are among the most commonly reported parasitic infections in NHPs all over the world. The strongylid species are indistinguishable through traditional microscopic approaches; molecular analyses have recently led to the identification, at the species level, of *Trichostrongylus colubriformis* larvae/eggs from ring-tailed lemurs in captivity [45]). DNA metabarconding has been used for studying strongylid community diversity in the wild [46], assuming the presence of multi-species infections in that context. A similar approach may also find application in captive NHPs in the future, overcoming the limitations of classic PCRs when dealing with a mixture of different DNAs. 

*Strongyloides* spp. infections are commonly reported in free-ranging and captive NHPs in many countries [47]. Three *Strongyloides* species have been described in NHPs, two of which are potentially zoonotic (*S. stercoralis* and *S. fuelleborni*). Strongyloidiasis by *S. stercoralis* is present in humans and dogs in Italy [48,49,50] and *S. fuelleborni* eggs were described by copromicroscopic exam in the feces of baboons in a zoo in southern Italy [28]. The molecular approach is important for distinguishing species with and without zoonotic potential, when NHPs and humans share the same environment such as in captive and semi-captive settings [47,51]. 

To date, four species of *Cryptosporidium* have been detected in NHPs, with the bulk of the isolates from captive NHPs belonging to zoonotic genotypes of *C. hominis* [2,3,4]. The prevalence of *Cryptosporidium* spp. seems to be low in zoological gardens, with values varying from 0% in zoos in Cordoba and Almuñecar in Spain [52,53] to 1% in French zoos [54]. A much higher prevalence was previously reported in a Barcelona zoo, with 27.6-44.4% of animals positive over a ten-year period [55,56]. A high prevalence (66.7%) of *Cryptosporidium* sp. was also reported in the fecal samples of NHPs from two zoo in Southern Italy [28]. High hygiene standards and the management of water sources in zoos probably account for the control of *Cryptosporidium* in modern zoos. 

*Giardia* was reported to have a moderate prevalence among all investigated groups except apes, with a prevalence varying from 20% in samples from OWM to 40.5% in NWM. Apes are actually the only group of animals spatially isolated from other NHPs. Wether this is due to casualty, given the low number of sampled animals, or to a minor transmission risk could be a matter of investigation. A moderate to high prevalence of *Giardia* infections has been reported in NHPs in European zoos, varying from 28% at Zagreb zoo [57] to 47% of lemurs kept in Rome zoo [30] and 70% in two Spanish zoos [58]. A more recent, comprehensive survey on protist infections in Cordoba zoo reported that 22% of NHPs were infected with *Giardia* [53]. All surveys report asymptomatic infections, in agreement with this survey. The presence of visible *Giardia* spp. cysts at copromicroscopic examination only in one sample and the high number of qPCR-positive samples with ct > 30 may indicate infections with a low burden in the animals. Alternatively, chronic or repeated infections, as reflected by several positive results throughout the year in the same enclosure, may explain immune system activation towards the protozoan, resulting in asymptomatic infections [59]. 

Sequences of good quality could be retrieved only from seven samples, and none could be sequenced at more than one locus. An analysis of triosephosphate isomerase (TPI) or β-giardine (BG) genes attributed all isolates to subassemblage BIV. The dominance of assemblage B in NHPs has already been reported [6], and this assemblage seems to be well-adapted to all primates [8]. Sub-assemblage BIV has been widely reported in NHPs in captivity in Europe [30,57,58,60] and outside Europe [61]. With the exception of subassemblage AIII, assemblage A and B are all considered zoonotic, and the BIV sub-assemblage in particular is widespread among humans also in Europe [8,10]. Transmission from humans to animals in a zoological facility seems a quite unrealistic eventuality, but common hygienic procedures followed in zoos do not exclude the opposite route. The sanitary risk should thus be taken into account by keepers and zoo staff and quantified by performing regular testing for infections with this protozoan in NHPs. 

In total, 80.7% of animal groups tested positive for *Blastocystis* at least once throughout the sampling period, confirming this as the most common eukaryotic protist in the intestinal tract of captive NHPs, with the reported prevalence reaching 45.5% in Cordoba zoo, 66.6% in Almuneçar zoo, and 20.3% in a survey among six European zoos [52,53,62]. Molecular studies of NHP isolates are often carried out to trace, in some cases, the sources of infection, and especially to investigate the eventuality of a human-to-animal or animal-to-human transmission. Indeed, not surprisingly, NHPs and humans are most often infected by the same subtypes, with ST1-3 being the most frequent in both [63,64]. The identification of the same alleles within subtypes in NHPs and their animal handlers has demonstrated that zoonotic transmission between the two is possible in zoos, in both directions [64]. In this survey, we did not have access to human samples, but another interesting pathway of *Blastocystis* transmission involving synantropic rodents can be hypothesized. ST4 is indeed considered rare in NHPs [63,65], being isolated in a few studies in *Varecia rubra* and captive lemurs [63,65,66], but it is the most frequent in rodents [50,61]; in particular, the allelic combination shared by both NHPs and rats in this study has been repeatedly isolated from rodents [53,67,68,69,70]. Because ST4, with the same allelic composition, has also been isolated from humans [71], and because ST4 has been demonstrated to have a higher correlation with the occurrence of symptomatic infections in humans [72], the zoonotic transmission of *Blastocystis* isolates from animals to zoo keepers in the context of zoos should not be overlooked.

## 5. Conclusions

In conclusion, this parasitological survey showed a generally low prevalence and burden of gastrointestinal helminth species in captive NHPs and more widespread infections by protist species in both facilities. 

Rodents were found to represent a source of environmental fecal contamination, likely responsible for the transmission of both helminthic (*H. diminuta*) and protist taxa (*Blastocystis* ST4) to NHPs, different from what was recently reported in another survey in Spain [53]. Rodent control thus becomes pivotal in avoiding parasitic disease spread in NHP collections.

Given that the zoonotic potential of the isolated taxa has been proven or not ruled out, it is critical that zoo staff caring for primates maintain good hygiene practices. Routine copromicroscopic testing and targeted treatments are recommended to keep a low parasite load in the collections, reducing the impact of parasites on the health of captive NHPs, as well as the risk of zoonotic transmission to the zoo staff.

## Figures and Tables

**Table 3:** Parasites in the animal feces, as detected by copromicroscopy and molecular analyses.

			Copromicroscopic Analyses					Molecular Analyses			
Host		Tested Samples	Tric.	Cap.	Str.	H.d.	*Giardia* spp.	*Giardia duodenalis*	*Giardia* Assemblages	*Blastocystis* sp.	*Blastocystis* Subtypes
NWM	animal groups	12	1	6	3	1	Neg	9	B	11	ST4, ST8
	feces	42	1	6	3	1	Neg	17		16	
OWM	animal groups	5	2	1	Neg	1	Neg	3	B	1	nd
	feces	20	5	3	Neg	1	Neg	4		3	
Lemurs	animal groups	8	3	Neg	2	Neg	1	4	B	8	ST4
	feces	30	6	Neg	5	Neg	1	11		22	
Apes	animal groups	1	Neg	Neg	Neg	Neg	Neg	Neg	-	1	nd
	feces	4	Neg	Neg	Neg	Neg	Neg	Neg		1	

NWM = New World Monkeys; OWM = Old World Monkeys; Tric. = *Trichuris* spp.; Cap. = *Capillariinae* Gen. spp. sp.; Str. = strongylid eggs, *Strongyloides* spp.; H.d. = *Hymenolepis diminuta*; Neg = negative; nd = not determinated.

## Data Availability

The original contributions presented in the study are included in the article; further inquiries can be directed to the corresponding authors.

## Supplementary Materials

The following supporting information can be downloaded at: https://www.mdpi.com/article/10.3390/ani14172607/s1, Table S1: List of captive primates species included in the study.

## References

[1] Xiao L., Fayer R. (2008). Molecular characterization of species and genotypes of *Cryptosporidium* and *Giardia* and assessment of zoonotic transmission. Int. J. Parasitol..

[2] Feng Y. (2010). *Cryptosporidium* in wild placental mammals. Exp. Parasitol..

[3] Li W., Kiulia N.M., Mwenda J.M., Nyachieo A., Taylor M.B., Zhang X. (2011). *Cyclospora papionis*, *Cryptosporidium hominis*, and human-pathogenic *Enterocytozoon bieneusi* in captive baboons in Kenya. J. Clin. Microbiol..

[4] Ye J., Xiao L., Ma J., Guo M., Liu L., Feng Y. (2012). Anthroponotic Enteric Parasites in Monkeys in Public Park, China. Emerg. Infect. Dis..

[5] Feng Y., Ryan U.M., Xiao L. (2018). Genetic Diversity and Population Structure of *Cryptosporidium*. Trends Parasitol..

[6] Ryan U., Cacciò S.M. (2013). Zoonotic potential of *Giardia*. Int. J. Parasitol..

[7] Johnston A.R., Gillespie T.R., Rwego I.B., McLachlan T.L., Kent A.D., Goldberg T.L. (2010). Molecular epidemiology of cross-species *Giardia duodenalis* transmission in western Uganda. PLoS Negl. Trop. Dis..

[8] Sprong H., Cacciò S.M., van der Giessen J.W.B., ZOOPNET Network and Partners (2009). Identification of Zoonotic Genotypes of *Giardia duodenalis*. PLoS Negl. Trop. Dis..

[9] Levecke B., Geldhof P., Claerebout E., Dorny P., Vercammen F., Cacciò S.M., Vercruysse J., Geurden T. (2009). Molecular characterisation of *Giardia duodenalis* in captive non-human primates reveals mixed assemblage A and B infections and novel polymorphisms. Int. J. Parasitol..

[10] Brynildsrud O., Tysnes K.R., Robertson L.J., Debenham J.J. (2018). *Giardia duodenalis* in primates: Classification and host specificity based on phylogenetic analysis of sequence data. Zoonoses Public Health.

[11] Hublin J.S.Y., Maloney J.G., Santin M. (2021). *Blastocystis* in domesticated and wild mammals and birds. Res. Vet. Sci..

[12] Ilík V., Kreisinger J., Modrý D., Schwarz E.M., Tagg N., Mbohli D., Nkombou I.C., Petrželková K.J., Pafčo B. (2023). High diversity and sharing of strongylid nematodes in humans and great apes co-habiting an unprotected area in Cameroon. PLoS. Negl. Trop. Dis..

[13] Barda B.D., Rinaldi L., Ianniello D., Zepherine H., Salvo F., Sadutshang T., Cringoli G., Clementi M., Albonico M. (2013). Mini-FLOTAC, an innovative direct diagnostic technique for intestinal parasitic infections: Experience from the field. PLoS Negl. Trop. Dis..

[14] Cassini R., Parraga M.A., Signorini M., Frangipane di Regalbono A., Sturaro E., Rossi L., Ramanzin M. (2015). Lungworms in Alpine Ibex (*Capra ibex*) in the Eastern Alps, Italy: An ecological approach. Vet. Parasitol..

[15] Modrý D., Pafčo B., Petrželková K.J., Hasegawa H. (2018). Parasites of Apes an Atlas of Coproscopic Diagnostics.

[16] Vonfeld I., Prenant T., Polack B., Guillot J., Quintard B. (2022). Gastrointestinal parasites in non-human primates in zoological institutions in France. Parasite.

[17] Galuppi R., Piva S., Castagnetti C., Iacono E., Tanel S., Pallaver F., Fioravanti M.L., Zanoni R.G., Tampieri M.P., Caffara M. (2015). Epidemiological survey on *Cryptosporidium* in an Equine Perinatology Unit. Vet. Parasitol..

[18] Ortolani E.L. (2000). Standardization of modified Ziehl-Neelsen technique to stain oocysts of *Cryptosporidium* sp. Rev. Bras. Parasitol. Vet..

[19] Miller W.A., Gardner I.A., Atwill E.R., Leutenegger C.M., Miller M.A., Hedrick R.P., Melli A.C., Barnes N.M., Conrad P.A. (2006). Evaluation of methods for improved detection of *Cryptosporidium* spp. in mussels (*Mytilus californianus*). J. Microbiol. Meth..

[20] Verweij J.J., Schinkel J., Laeijendecker D., van Rooyen M.A., van Lieshout L., Polderman A.M. (2003). Real-time PCR for the detection of *Giardia lamblia*. Mol. Cell. Probes..

[21] Lalle M., Jimenez-Cardosa E., Cacciò S.M., Pozio E. (2005). Genotyping of *Giardia duodenalis* from humans and dogs from Mexico using a beta-giardin nested polymerase chain reaction assay. J. Parasitol..

[22] Sulaiman I.M., Fayer R., Bern C., Gilman R.H., Trout J.M., Schantz P.M., Das P., Lal A.A., Xiao L. (2003). Triosephosphate isomerase gene characterization and potential zoonotic transmission of *Giardia duodenalis*. Emerg. Infect. Dis..

[23] Stensvold C.R., Ahmed U.N., Andersen L.O., Nielsen H.V. (2012). Development and evaluation of a genus-specific, probe-based, internal-process-controlled real-time PCR assay for sensitive and specific detection of *Blastocystis* spp. J. Clin. Microbiol..

[24] Scicluna S.M., Tawari B., Clark C.G. (2006). DNA Barcoding of *Blastocystis*. Protist.

[25] Altschul S.F., Gish W., Miller W., Myers E.W., Lipman D.J. (1990). Basic local alignment search tool. J. Mol. Biol..

[26] Jolley K.A., Bray J.E., Maiden M.C.J. (2018). Open-access bacterial population genomics: BIGSdb software, the PubMLST.org website and their applications. Wellcome Open Res..

[27] Almeida Lourenco M.F. (2023). Survey of Gastrointestinal Parasites of Non-Human Primates from Two Iberian Zoos and Perspectives of Their Integrated Control. Ph.D. Thesis.

[28] Fagiolini M., Lia R.P., Laricchiuta P., Cavicchio P., Mannella R., Cafarchia C., Otranto D., Finotello R., Perrucci S. (2010). Gastrointestinal parasites in mammals of two Italian zoological gardens. J. Zoo. Wildl. Med..

[29] Rushmore J., Bisanzio D., Gillespie T.R. (2017). Making New Connections: Insights from Primate-Parasite Networks. Trends Parasitol..

[30] Berrilli F., Prisco C., Friedrich K.G., Di Cerbo P., Di Cave D., De Liberato C. (2011). *Giardia duodenalis* assemblages and *Entamoeba* species infecting non-human primates in an Italian zoological garden: Zoonotic potential and management traits. Parasit. Vectors..

[31] Rondón S., Cavallero S., Montalbano Di Filippo M., De Liberato C., Berrilli F., Capitani N., D’Amelio S. (2024). Intestinal parasites infecting captive non-human primates in Italy. Front. Vet. Sci..

[32] Galoș F., Anghel M., Ioan A., Ieșanu M.I., Boboc C., Boboc A.A. (2022). *Hymenolepis diminuta* Infection in a Romanian Child from an Urban Area. Pathogens.

[33] Yao C. (2023). Human infections by the rat tapeworm *Hymenolepis diminuta* in China. Trans. R. Soc. Trop. Med. Hyg..

[34] Marangi M., Zechini B., Fileti A., Quaranta G., Aceti A. (2003). *Hymenolepis diminuta* infection in a child living in the urban area of Rome, Italy. J. Clin. Microbiol..

[35] Guardone L., Macchioni F., Torracca B., Gabrielli S., Magi M. *Hymenolepis diminuta* (rat tapeworm) infection in a dog in Liguria, northwest Italy. Proceedings of the XXVI SoIPA (Società Italiana di Parassitologia) National Congress.

[36] D’Ovidio D., Noviello E., Pepe P., Del Prete L., Cringoli G., Rinaldi L. (2015). Survey of *Hymenolepis* spp. in pet rodents in Italy. Parasitol. Res..

[37] Cavallero S., Nejsum P., Cutillas C., Callejón R., Doležalová J., Modrý D., D’Amelio S. (2019). Insights into the molecular systematics of *Trichuris* infecting captive primates based on mitochondrial DNA analysis. Vet. Parasitol..

[38] Cavallero S., Montalbano Di Filippo M., Rondón S., Liberato C., D’Amelio S., Friedrich K.G., Berrilli F. (2020). Nuclear and Mitochondrial Data on *Trichuris* from *Macaca fuscata* Support Evidence of Host Specificity. Life.

[39] Rivero J., Cutillas C., Callejón R. (2021). *Trichuris trichiura* (Linnaeus, 1771) From Human and Non-human Primates: Morphology, Biometry, Host Specificity, Molecular Characterization, and Phylogeny. Front. Vet. Sci..

[40] Cavallero S., De Liberato C., Friedrich K.G., Di Cave D., Masella V., D’Amelio S., Berrilli F. (2015). Genetic heterogeneity and phylogeny of *Trichuris* spp. from captive non-human primates based on ribosomal DNA sequence data. Infect. Genet. Evol..

[41] Helenbrook W.D., Wade S.E., Shields W.M., Stehman S.V., Whipps C.M. (2015). Gastrointestinal Parasites of Ecuadorian Mantled Howler Monkeys (*Alouatta palliata aequatorialis*) Based on Fecal Analysis. J. Parasitol..

[42] Justine J.L. (1988). *Capillaria brochieri* n. sp. (Nematoda: Capillariinae) intestinal parasite of the chimpanzee (*Pan paniscus*) in Zaire. Ann. Parasitol. Hum. Comp..

[43] Pizzi R., Gordon J.C., Flach E.J., Routh A.D., Clark B., Boardman W.S. (2008). *Capillaria hepatica* (syn *Calodium hepaticum*) in primates in a zoological collection in the UK. Vet Rec..

[44] Fuehrer H.P. (2014). An overview of the host spectrum and distribution of *Calodium hepaticum* (syn. Capillaria hepatica): Part 2—Mammalia (excluding Muroidea). Parasitol. Res..

[45] Dini F.M., Caffara M., Galliani M., Cotignoli C., Capasso M., Tedesco P., Galuppi R. (2023). Unveiling a novel parasitosis: *Trichostrongylus colubriformis* infection in captive ring-tailed lemurs (*Lemur catta*). Int. J. Parasitol. Parasites Wildl..

[46] Pafčo B., Čížková D., Kreisinger J., Hasegawa H., Vallo P., Shutt K., Todd A., Petrželková K.J., Modrý D. (2018). Metabarcoding analysis of strongylid nematode diversity in two sympatric primate species. Sci. Rep..

[47] Nosková E., Sambucci K.M., Petrželková K.J., Červená B., Modrý D., Pafčo B. (2024). *Strongyloides* in non-human primates: Significance for public health control. Philos. Trans. R. Soc. Lond. B. Biol. Sci..

[48] Stancampiano L., Morandi F., Usai F., Benazzi C., Pietra M. (2011). An unusual case of fatal *Strongyloides stercoralis* hyperinfection. Veterinaria.

[49] Ottino L., Buonfrate D., Paradies P., Bisoffi Z., Antonelli A., Rossolini G.M., Gabrielli S., Bartoloni A., Zammarchi L. (2020). Autochthonous Human and Canine *Strongyloides stercoralis* Infection in Europe: Report of a Human Case in An Italian Teen and Systematic Review of the Literature. Pathogens.

[50] Paradies P., Iarussi F., Sasanelli M., Capogna A., Lia R.P., Zucca D., Greco B., Cantacessi C., Otranto D. (2017). Occurrence of strongyloidiasis in privately owned and sheltered dogs: Clinical presentation and treatment outcome. Parasit. Vectors..

[51] Pafčo B., Kreisinger J., Čížková D., Pšenková-Profousová I., Shutt-Phillips K., Todd A., Fuh T., Petrželková K.J., Modrý D. (2019). Genetic diversity of primate strongylid nematodes: Do sympatric nonhuman primates and humans share their strongylid worms?. Mol. Ecol..

[52] Pérez Cordón G., Hitos Prados A., Romero D., Sánchez Moreno M., Pontes A., Osuna A., Rosales M.J. (2008). Intestinal parasitism in the animals of the zoological garden “Peña Escrita” (Almuñecar, Spain). Vet. Parasitol..

[53] Köster P.C., Dashti A., Bailo B., Muadica A.S., Maloney J.G., Santín M., Chicharro C., Migueláñez S., Nieto F.J., Cano-Terriza D. (2021). Occurrence and Genetic Diversity of Protist Parasites in Captive Non-Human Primates, Zookeepers, and Free-Living Sympatric Rats in the Córdoba Zoo Conservation Centre, Southern Spain. Animals.

[54] Osman M., El Safadi D., Benamrouz-Vanneste S., Cian A., Moriniere R., Gantois N., Delgado-Viscogliosi P., Guyot K., Bosc S., Chabé M. (2017). Prevalence, transmission, and host specificity of *Cryptosporidium* spp. in various animal groups from two French zoos. Parasitol. Res..

[55] Gomez M.S., Gracenea M., Gosalbez P., Feliu C., Enseñat C., Hidalgo R. (1992). Detection of oocysts of *Cryptosporidium* in several species of monkeys and in one prosimian species at the Barcelona zoo. Parasitol. Res..

[56] Gómez M.S., Torres J., Gracenea M., Fernandez-Morán J., Gonzalez-Moreno O. (2000). Further report on *Cryptosporidium* in Barcelona zoo mammals. Parasitol. Res..

[57] Beck R., Sprong H., Bata I., Lucinger S., Pozio E., Cacciò S.M. (2011). Prevalence and molecular typing of *Giardia* spp. in captive mammals at the zoo of Zagreb, Croatia. Vet. Parasitol..

[58] Martínez-Díaz R.A., Sansano-Maestre J., Martínez-Herrero M.d.C., Ponce-Gordo F., Gómez-Muñoz M.T. (2011). Occurrence and genetic characterization of *Giardia duodenalis* from captive nonhuman primates by multi-locus sequence analysis. Parasitol. Res..

[59] Dixon B.R. (2021). *Giardia duodenalis* in humans and animals—Transmission and disease. Res. Vet. Sci..

[60] Mravcová K., Štrkolcová G., Mucha R., Goldová M. (2021). Zoonotic assemblages of *Giardia duodenalis* in captive non-human primates from the largest zoo in Slovakia. J. Parasit. Dis..

[61] Karim M.R., Rongjun Y.F., Li T., Dong H., Li D., Zhang L., Li J., Jian F., Zhang S., Islam R.F. (2015). Multi-locus analysis of *Giardia duodenalis* from nonhuman primates kept in zoos in China: Geographical segregation and host-adaptation of assemblage B isolates. Infect. Genet. Evol..

[62] Köster P.C., Martínez-Nevado E., González A., Abelló-Poveda M.T., Fernández-Bellon H., de la Riva-Fraga M., Marquet B., Guéry J.P., Knauf-Witzens T., Weigold A. (2022). Intestinal Protists in Captive Non-human Primates and Their Handlers in Six European Zoological Gardens. Molecular Evidence of Zoonotic Transmission. Front. Vet. Sci..

[63] Stensvold C.R., Alfellani M.A., Nørskov-Lauritsen S., Prip K., Victory E.L., Maddox C., Nielsen H.V., Clark C.G. (2009). Subtype distribution of *Blastocystis* isolates from synanthropic and zoo animals and identification of a new subtype. Int. J. Parasitol..

[64] Parkar U., Traub R.J., Vitali S., Elliot A., Levecke B., Robertson I., Geurden T., Steele J., Drake B., Thompson R.C. (2010). Molecular characterization of *Blastocystis* isolates from zoo animals and their animal-keepers. Vet Parasitol..

[65] Alfellani M.A., Jacob A.S., Perea N.O., Krecek R.C., Taner-Mulla D., Verweij J.J., Levecke B., Tannich E., Clark C.G., Stensvold C.R. (2013). Diversity and distribution of *Blastocystis* sp. subtypes in non-human primates. Parasitology.

[66] Santín M., Gómez-Muñoz M.T., Solano-Aguilar G., Fayer R. (2011). Development of a new PCR protocol to detect and subtype *Blastocystis* spp. from humans and animals. Parasitol. Res..

[67] Mohammadpour I., Bozorg-Ghalati F., Gazzonis A.L., Manfredi M.T., Motazedian M.H., Mohammadpour N. (2020). First molecular subtyping and phylogeny of *Blastocystis* sp. isolated from domestic and synanthropic animals (dogs, cats and brown rats) in southern Iran. Parasit. Vectors.

[68] Mohammad N.A., Al-Mekhlafi H.M., Anuar T.S. (2018). Subtype distribution of *Blastocystis* isolated from humans and associated animals in an indigenous community with poor hygiene in Peninsular Malaysia. Trop. Biomed..

[69] Katsumata M., Yoshikawa H., Tokoro M., Mizuno T., Nagamoto T., Hendarto J., Asih P.B.S., Rozi I.E., Kimata I., Takami K. (2018). Molecular phylogeny of *Blastocystis* isolates from wild rodents captured in Indonesia and Japan. Parasitol. Res..

[70] Cian A., El Safadi D., Osman M., Moriniere R., Gantois N., Benamrouz-Vanneste S., Delgado-Viscogliosi P., Guyot K., Li L.-L., Monchy S. (2017). Molecular Epidemiology of *Blastocystis* sp. in Various Animal Groups from Two French Zoos and Evaluation of Potential Zoonotic Risk. PLoS ONE.

[71] Marangi M., Boughattas S., De Nittis R., Pisanelli D., Delli Carri V., Lipsi M.R., La Bella G., Serviddio G., Niglio M., Lo Caputo S. (2023). Prevalence and genetic diversity of *Blastocystis* sp. among autochthonous and immigrant patients in Italy. Microb. Pathog..

[72] Mattiucci S., Crisafi B., Gabrielli S., Paoletti M., Cancrini G. (2016). Molecular epidemiology and genetic diversity of *Blastocystis* infection in humans in Italy. Epidemiol. Infect..

