# Supplementary material for: Gastrointestinal Parasites in Non-Human Primates in Zoological Gardens in Northern Italy
Source: Animals (Basel). 2024 Sep 7;14(17):2607. doi: 10.3390/ani14172607 (PMC11394357; doi:10.3390/ani14172607)
Supplement: Supplementary file 1 [file animals-14-02607-s001.zip › animals-3170860-supplementary.pdf]

Table S1. List of captive primates species included in the study.

	N groups	N samples per group	Zoo
**Plathyrrini – New World Monkeys**			
Cebus sp.	1	4	ZG1
Sapajus apella	1	4	ZG2
Saimiri sciureus	1	2	ZG1
Saimiri boliviensis	2	4+1	ZG2
Atele paniscus	1	3	ZG1
Leontopithecus chrysomelas	1	4	ZG2
Saguinus bicolor	1	4	ZG2
Saguinus aedipus	1	4	ZG2
Callithrix penicillata	1	4	ZG2
Callithrix jacchus	1	4	ZG2
Callithrix argentata	1	4	ZG2
**Lemuridae – Prosimians**			
Varecia variegata	2	4+4	ZG1, ZG2
Varecia rubra	2	4+4	ZG1, ZG2
Eulemur albifrons	1	4	ZG1
Lemur catta	3	4+2+4	ZG1, ZG2
**Cercopithecidae – Old World Monkeys**			
Colobus guereza	2	4+4	ZG1, ZG2
Macaca mulatta	1	4	ZG1
Chlorocebus aethiops	1	4	ZG2
Mandrillus sphinx	1	4	ZG2
**Hominoidea – Apes**			
Hylobates lar	1	4	ZG1
